# Being Deaf in Mainstream Schools: The Effect of a Hearing Loss in Children’s Playground Behaviors

**DOI:** 10.3390/children9071091

**Published:** 2022-07-21

**Authors:** Brenda M. S. Da Silva, Carolien Rieffe, Johan H. M. Frijns, Herédio Sousa, Luísa Monteiro, Guida Veiga

**Affiliations:** 1 Department of Educational and Developmental Psychology, Leiden University, 2333 Leiden, The Netherlands; crieffe@fsw.leidenuniv.nl; 2 Department of Human Media Interaction, University of Twente, 7522 Enschede, The Netherlands; 3 Department of Psychology and Human Development, University College London, London WC1E 6BT, UK; 4 Department of Otorhinolaryngology and Head & Neck Surgery, Leiden University Medical Center, 2333 Leiden, The Netherlands; j.h.m.frijns@lumc.nl; 5 Leiden Institute for Brain and Cognition, Leiden University Medical Center, 2333 Leiden, The Netherlands; 6 Departamento de Otorrinolaringologia, Centro Hospitalar de Lisboa Central, 1169-045 Lisboa, Portugal; herediosousa@gmail.com; 7 Unidade de Otorrinolaringologia, Hospital Lusíadas Lisboa, 1500-458 Lisboa, Portugal; luisamonteiro.ent@gmail.com; 8 Departamento de Desporto e Saúde, Escola de Saúde e Desenvolvimento Humano, Universidade de Évora, 7004-516 Évora, Portugal; gveiga@uevora.pt; 9 Comprehensive Health Research Center (CHRC), Universidade de Évora, 7004-516 Évora, Portugal

**Keywords:** deaf and hard of hearing, preschool, social levels, play, observations, physical play, exercise play, inclusion

## Abstract

Naturalistic playground observations are a rich source of information when studying the social interactions of preschool children. On the playground, children can interact with their peers, explore different places and activities, and engage in different types of play. For deaf and hard of hearing (DHH) children, interactions at a playground can be more difficult because of the large number of auditory stimuli surrounding them. Constraints in the access to the social world on the playground might hamper DHH children’s interactions with their typically hearing (TH) peers, activities, and play. This pilot study aimed to examine the playground behaviors of preschool DHH children across three aspects: social levels, type of activities, and play choices. For this purpose, 12 preschool DHH children were observed during recess time, and their behaviors were coded and compared to their 85 TH peers. The preliminary findings indicate that DHH children spend less time in social interactions compared to their TH peers and that they still face difficulties when socially engaging with their TH peers. These findings suggest that interventions should focus on three aspects: the physical environment awareness of TH peers about communicating with DHH children, and the use of exercise play to facilitate social interactions between DHH children and their TH peers.

## 1. Introduction

A rich way to understand preschool-aged children’s skills and social interactions is through observing their play and peer interactions on playgrounds. Through the observation of naturalistic interactions—without adult interference—one can understand each child’s particular interest while playing, the dynamics between peers, their positioning in the social group, and their skills to enter and maintain interactions [1,2,3]. However, playgrounds are often neglected as a means to study child development [4], and this kind of research is especially scarce regarding preschool Deaf and Hard of Hearing (DHH) children. Most research in this field dates from 20 to 40 years ago (see [5] for a review) and reports that DHH children show different behaviors on playgrounds compared to their typically hearing (TH) peers, suggesting that the constraints in accessing the social world affect their social interactions with peers [5]. However, recently, many developments have taken place in terms of rehabilitation and technology (e.g., cochlear implants) and in terms of education. Currently, most DHH preschool children attend mainstream settings, which implies that they are mainly surrounded by TH peers [6,7,8]. In this pilot study, we focus on describing the playground behaviors of DHH preschool children across three categories: social levels, types of activities, and types of play.

School playgrounds usually offer many opportunities for children to engage in various forms of social behaviors, which have been related to higher levels of social competence and lower levels of internalizing symptoms and peer problems [2,9]. On playgrounds, children can play games (e.g., football, hide and seek), rest, communicate with peers, explore, and most importantly, they can play. Play, defined as ‘a spontaneous, pleasurable, and self-guided activity’, provides children with a unique opportunity for their overall development [10,11]. Yet, communication seems to be a key aspect of playing with peers [12,13]. Thus, children who have hindered communication—such as DHH children—might face more challenges while playing. In fact, social play in itself, especially in the preschool years, is already difficult for these children to follow, as the interactions and rules keep changing, which might be more difficult for DHH children to follow [14]. Moreover, social play can become even more difficult when it occurs in the playground setting, with all the different auditory and kinetic stimuli [15]. In this chaotic setting, DHH children might miss out on information that is necessary to engage and maintain play with their peers, thus impacting their play choices [5,16].

While on the playground, children can broadly show three types of behaviors: nonsocial (i.e., solitary and reticent), parallel, and/or social [9,17,18,19]. Social behaviors are those in which the child is communicating and/or playing with their peers without any specific organization or in a cooperative way [18,19,20].

Nonsocial behaviors are those in which children do not try to initiate or engage in interactions with their peers, even though peers are available [17]. Some children choose to play solitarily; however, the most frequent forms of nonsocial behaviors are reticent [21,22]. Reticent behaviors can be split into onlooking behaviors, which is when children observe their peers without attempting to join them, or unoccupied behaviors, which is when children wander around the playground with no specific focus or purpose [21,22,23]. Although there is no consensus regarding the adaptive function of solitary behavior, reticent behavior has been related to less emotional understanding, internalizing symptoms, lack of social skills, and peer rejection [3,24,25,26].

Parallel behaviors are those in which the child plays alone but is in close proximity with their peers, sometimes observing others and imitating them [19,20,27]. Parallel behaviors seem to mark the transition between nonsocial-solitary behaviors and social-group behaviors [19,28]. That is, children are interested—and also learning—about their peers, including their play preferences, their interests, how they respond to situations, and how they engage [29,30]. Therefore, parallel behaviors provide children with a unique opportunity to learn about their peers and to understand how to initiate interactions with them and maintain them [29].

Access to their peers and joining in social play might be more challenging for DHH children. Studies show that DHH children are less successful in initiating and maintaining social interactions with their peers [7,13,31]. DHH children thus spend more time alone at playgrounds compared to their TH peers [32], whilst showing more onlooking behaviors [33]. Moreover, when they interact, their interactions are shorter compared to their TH peers, which makes their social networks more fragile [29,34]. Note that their TH peers might form a barrier here, as DHH children are less often invited or allowed to join in play [7,13,32], and TH peers are overall less willing to initiate interactions with DHH children [31].

Regarding play, there are two major forms: physical and pretend play [11,35]. Physical play can be characterized by moderate to vigorous physical activity that can be split into: exercise play (e.g., running, climbing) and rough-and-tumble play (e.g., chasing, play-fighting, etc.) [35]. Pretend play can be characterized by a symbolic—and playful—conversion of something into something else, which can be split into role play (i.e., when an object is converted into something else) and fantasy play (i.e., impersonating someone else) [36,37,38]. The research focused on the play behaviors of DHH children shows that DHH children seem to avoid pretend play (e.g., [13,39,40]. In fact, recent studies have shown that DHH children engage more in physical play rather than pretend play [41,42].

Using naturalistic playground observations, the aim of this pilot study is to describe the spontaneous playground behaviors of DHH preschoolers integrated into mainstream education and compare them to their TH peers. Within the umbrella of playground behaviors, we focus on social levels, type of activities, and type of play that these children engage in during recess time. Past studies looked into play choices, and the social levels of preschool DHH children (see [5] for a review); however—to the best of our knowledge—no previous studies have also looked into the engagement in different types of activities. Focusing on these three aspects in a single study (i.e., social levels, type of activities, and play choices) allows us to achieve a more in-depth understanding of how preschool DHH children behave on playgrounds. Regarding the social levels and based on previous research [33], we expect that DHH children show more nonsocial behaviors—in particular onlooking ones and less social behaviors—than their TH peers. Regarding the type of activities, due to the lack of previous research, no assumptions are made. Regarding the play choices, based on previous research [41,42], we expect that DHH children engage more frequently in physical play than any other type of play. These preliminary findings might provide a more comprehensive view of how hearing loss can influence social participation and play preferences of DHH children in mainstream education.

## 2. Materials and Methods

### 2.1. Participants and Procedures

A total of 12 DHH children (Mage = 59.67 months, SD = 8.60 months; 58% boys) participated in this study. Six DHH children had a profound loss and bilateral cochlear implants; four had a severe loss and conventional hearing aids (HA); and two children had a severe loss and HA in the left ear and a profound loss and CI in the right ear. All of the DHH children in our study used spoken language as their primary mode of communication. Similar to previous studies [34,43], the classmates of our DHH participants were recruited as the control group. DHH children and their parents were recruited in two hospitals in Lisbon and asked to participate in this study during their hospital visit. The DHH children’s parents were asked for the name of the preschool that their child attended, and only when the parents gave their permission was the preschool then contacted at a later stage to participate. The preschools were first asked for their participation, and when they agreed, parents of all children in the group or class with the DHH child were also asked to participate. A total of 85 TH children (Mage = 61.98 months, SD = 11.42 months; 45% boys) participated in the study. Parents, preschools, and hospitals were informed about the goals and procedures of the study, how data would be handled and stored to guarantee the participants’ privacy, and the voluntary nature of their participation. The parents also gave written consent. The children were informed about the purpose of the study and gave verbal consent to their own participation. Approval for the study was obtained from the ethics committees of all institutions involved.

### 2.2. Materials

#### Playground Observations

A total of 548 videos were collected and randomly distributed between two trained observers. To test inter-observer reliability, 24% of the videos were double-coded. Each child’s behavior at outdoor recess was randomly video recorded over 3 days. Each 3-min video was split into 12 segments (15 s each), in which the observer identified the prevalent type of behavior (social level; type of activity; type of play). A frequency score for each type of behavior was obtained through the division of the total number of segments in which the child was involved in each specific type of behavior by the segments in which the child was observed. If children were not seen during any segment, their behaviors were coded as “not observed”.

Social level (k = 0.91) was coded based on social levels categories suggested by Rubin [18], that is, unoccupied, onlooking, solitary, parallel, and social. A nonsocial category was obtained through the sum of unoccupied, onlooking, and solitary behaviors.

The type of activity (k = 0.88) was coded based on the different activities that children engaged in on the playground, that is, play; games with rules; talking; exploratory behaviors; transitioning between activities; personal care; aggressive behaviors; and other behaviors. If the children were not engaged in any activity, their behavior was coded as reticent.

Play behaviors (k = 0.89) were coded according to the observation scheme developed by Veiga et al. [2]. The prevalent type of play in which the child was engaging during each segment was coded (i.e., exercise play; rough-and-tumble; fantasy play, role play; constructive play; playing with the equipment; or other play).

### 2.3. Statistical Analyses

First, the descriptive data were gathered for both groups. Second, group differences were assessed for each variable. Due to the non-normality of the data, we used the Mann–Whitney U test to assess group differences. Third, the within-group differences were assessed through Friedman’s rank test, and a post hoc comparison between social levels, type of activities, and the types of play of each group were assessed through pairwise Wilcoxon sum rank tests. The within-group’s differences of social levels were assessed considering the ‘nonsocial’, ‘parallel’, and ‘social’ categories. While assessing in-group differences, the frequency of “Not observed” episodes was not considered for the analysis, as they do not represent behaviors.

## 3. Results

As reported in Table 1, differences between the groups appeared regarding the social levels of interaction at the playground during recess time. Compared to their TH peers, DHH children showed fewer social interactions (U = 230.50, *p* = 0.002), but more nonsocial behaviors (U = 242, *p* = 0.003). Within the domain of nonsocial behaviors, DHH children showed more onlooking behaviors (U = 265, *p* = 0.007) and solitary behaviors (U = 281, *p* = 0.012) than their TH peers. Parallel interactions were almost absent for children in both groups.

A within-group comparison of the level of social interactions showed no difference within the DHH group between social and nonsocial play (Z = −1.26, *p* = 0.209), whereas TH children engaged most in social play (Z = −7.68, *p* < 0.001). Furthermore, both groups engaged less in parallel interactions, in comparison to social (DHH: Z = −3.06, *p* = 0.002; TH: Z = −7.85, *p* < 0.001), and nonsocial interactions (DHH: Z = −2.93, *p* = 0.003; TH: −8.01, *p* < 0.001).

No differences were found between groups regarding the engagement in different types of activities (Table 1). Within-group comparisons of the type of activity (Table 2), showed that the DHH children equally preferred to play, and communicate over the remaining activities, whereas TH children preferred play over all the remaining activities. For TH children, communication was the second preferred activity (Table 2). Furthermore, reticent behaviors were the third most prevalent activity for both groups (Table 2).

Regarding play, differences between the groups were found for exercise play. Compared to their TH peers, DHH children engaged more in exercise play (U = 299, *p* = 0.026) (Table 1). Within-group comparisons showed that both groups preferred exercise play over all the remaining types of play (Table 3). Furthermore, the DHH children preferred to engage in constructive play with equipment rather than fantasy play (Z = −2.50, *p* = 0.012), whilst TH children preferred both forms of pretend play over constructive play (Table 3).

## 4. Discussion

The main purpose of our pilot study was to describe the spontaneous playground behaviors (i.e., social levels, type of activities, and play choices) of preschool DHH children who attend mainstream preschools and compare them to their TH peers. As previously mentioned, past studies have focused on certain aspects of the playground behaviors of preschool DHH children. However, these studies were mostly focused on children with conventional hearing aids or who were implanted later, whereas neonatal hearing screening at the national level, the development of new technologies, and early interventions now provide much better opportunities for DHH children to improve their hearing compared to 20 years ago (see [5] for a review). Thus, understanding the differences—or similarities—between the current and past results might allow us to reflect on how the progress in the rehabilitation of DHH children has influenced their behaviors and abilities to participate within the peer group. Contrary to previous studies, the DHH children did not spend most of their recess time at the school’s playground in nonsocial behaviors, but the prevalence of these nonsocial behaviors is still significantly higher than for their TH peers. Unfortunately, preliminary findings of the present study also suggest that DHH children still face difficulties in engaging in social interactions with their TH peers and that they maintain similar patterns of interaction as found in studies from more than 2 decades ago [5].

In line with previous studies [32,33], the DHH children in our study engaged less frequently in social interactions and more frequently in nonsocial behaviors compared to their TH peers. Within the TH group, the children preferred to engage in social interactions rather than nonsocial behaviors; however, for DHH children, this preference was not confirmed. The DHH children seemed to be equally engaged in both types of behaviors (social vs. nonsocial). Moreover, DHH children spent more time in onlooking behaviors compared to their TH peers [32,33]. That is, DHH children spent time in proximity with their peers, observing them without attempting to join them. These findings are often interpreted in regards to problems in the socio-emotional development of DHH preschoolers, such as a lack of emotional understanding [44] and emotion regulation [45], which may hinder them from freely participating in social interactions with their TH peers.

In our study, all of the DHH children used spoken language as their primary mode of communication, similar to their TH peers. To date, this is the case for most DHH children who have no additional diagnoses because most early intervention programs are focused on spoken language acquisition [46]. Although technological advances in hearing devices allow DHH children to receive better auditory input, their hearing is still not comparable to that of their TH peers, and many factors can influence their communication in a predominantly TH world. Recent studies show that despite sharing the same communication mode, good language skills, compatible socio-emotional functioning, and good social skills, DHH children are still more often ignored or excluded by their DHH peers [47]. Various factors may cause this lack of social inclusion. For example, poor acoustics of the playground might hinder full participation or any participation at all. Yet, this lack of social inclusion might also be partially caused by a lack of awareness in TH children to understand what it takes to include a DHH child in a noisy environment, such as playgrounds, in play. For example, the DHH child has to see where sounds are coming from and who is talking, and additionally, the face of the speaker should be facing the light. Furthermore, TH children should wait until one is finished talking before starting to talk. In sum, TH children have to be aware that DHH children rely on visual cues to facilitate their access to social information. However, all these tasks are not easy for young children on the playgrounds. Moreover, poor acoustics of the playground might further hinder full participation or any participation at all. However, disregarding the access to sound, children can also easily exclude children who are perceived as “different”, i.e., purposely ignoring or excluding a DHH child. All of these facets might further contribute to difficulties for DHH children to develop their socio-emotional skills in ways that TH children do: in naturalistic, spontaneous settings with their peers.

In our study, the engagement of children from both groups in parallel play was almost absent. Although parallel play seems to play an important role in the transition from solitary play to social-group play, previous studies have referred that this stage is not obligatory and that they only arise if/when children need them to facilitate interactions, which might explain our results [28,48].

When on the playground, DHH and TH preschoolers show similar activities engagement: they spent most of their time playing and communicating. Previous studies have suggested that because of their communication difficulties, DHH children might avoid communication with their peers in playground settings, which can also hinder their engagement in play (see [5] for a review). Our current findings suggest that advances in technology and rehabilitation might have positively contributed to their opportunities for communication in the mainstream setting. There are different types of hearing aids, but as a whole, all of these devices have been improved over the years. Furthermore, nowadays, cochlear implants are a commonly used device for people who have severe to profound hearing loss, which allows them to have access to a wider range of auditory information compared to conventional hearing aids [49]. Moreover, in our study, two-thirds of the DHH children had CI’s, which might explain the positive results regarding communication. When comparing a DHH child with a CI with a child with a conventional hearing aid, or no hearing aid at all, within the same range of hearing loss, the CI child will be able to access more auditory information and, therefore, their communication abilities will be closer to that of TH children [49]. In fact, after implantation, these children can achieve better communication skills and therefore interact more and take more advantage of the auditory clues provided by the social environment [16], which might explain our results.

Regarding the engagement in play, our results are in line with the recent literature [41,42] that shows that DHH children prefer exercise play over any other type of play. Our hypothesis that DHH children would prefer physical play over pretend play was also confirmed. When comparing the two types of play—physical and pretend—pretend play requires more complex language abilities, better communication, and social skills [50,51]. Therefore, our results are in line with previous studies that show that DHH children tend to avoid this type of play because it is more demanding in terms of language and socio-emotional skills [51,52]. In comparison, exercise play might be less demanding in terms of language, and emotional skills, making it more attractive for DHH children [15]. Exercise play allows DHH children to use their bodies and movements (rather than words) to communicate, be in synchrony, and cooperate with their peers, promoting more frequent and more positive interactions [15]. Physical play, especially exercise play, has been suggested to provide DHH children with a more suitable alternative to engage in play with their peers [15]. Furthermore, contrary to their TH peers, DHH children preferred constructive play over pretend-fantasy play. The reasoning for this preference seems to be in line with the pretend vs. physical play preference. Previous studies that compared the engagement of DHH preschool children in both types of play showed that these children spend more time in constructive play rather than in pretend play [13,53]. Similar physical play, constructive play is less dependent on verbal communication in comparison to pretend play. Furthermore, constructive play does not require children to have a play partner and can be easily engaged alone. Indeed previous studies report that DHH children prefer to engage solitarily in constructive play rather than engage in cooperative/social types of play [53].

As a final note, we want to address four limitations that might be addressed in future studies. First, this is a pilot study with a small sample size. Future studies should replicate our study with a larger sample to confirm if our results are maintained with a more representative sample of the population. Furthermore, a larger sample size would allow comparisons between DHH children who use conventional hearing aids vs. those who use cochlear implants. Comparisons between both types of hearing devices can further inform us about the benefits of each type of hearing device and how each device singularly impacts social participation and the inclusion of DHH children within the peer group. Second, in the present study, all of the participating DHH children were in different classes or schools and, consequently, only had TH peers available for them at school. DHH children in mainstream schools still prefer to engage in interactions with similar peers [8], which might have influenced the high prevalence of nonsocial behaviors. It will be worthwhile that future studies focus on schools where DHH children have other DHH children, as well as TH peers. The presence of DHH peers on the playground might allow these children to feel more welcomed into engaging in group interactions with other DHH children. Third, all the DHH children in our study used spoken language as their primary mode of communication. However, this is not true for all DHH children mainly because spoken language is not accessible to them (e.g., they have irreversible deafness; they do not have criteria to benefit from hearing devices) or because they rely on a more visual—or bilingual—modes of communication (e.g., sign supported language) [54]. DHH children who do not use spoken language might have a different social experience—and engagement—than the DHH children included in our study. Therefore, future studies could also include DHH children who primarily use sign language, or sign-supported language, to understand how different communication modes influence the social participation of DHH children within the peer group. Fourth, it is important to highlight the importance of naturalistic observations as a method to obtain information regarding children’s interactions. Observations allow us to specifically understand what children are doing and with whom during the period of observation. However, this method is very time-consuming, and it only allows us to capture limited fragments of the children’s interactions. New studies that include new methodologies—such as sensor data—are also important for the study of playground behaviors, as they enable us to capture spontaneous behavior through intense and continuous data [2,3]. Future studies should combine observation data with these new technologies to better understand the social functioning of preschool DHH children in playground settings. Additionally, future studies should also include sociometric measures (e.g., likeability, friendship ratings, popularity), as these peer reports can further inform us about the social participation and social positioning of DHH children within the peer group.

## 5. Conclusions

The outcomes of our study provide a current—and extensive—picture of the playground behaviors of DHH children. Our results might increase the awareness of the positive impact that advancement in technology, rehabilitation, and educational policies have brought to these children. DHH children currently seem more prone to interact and communicate with their TH peers than DHH children 20 to 40 years ago. However, certain playground behaviors (i.e., high prevalence of onlooking behaviors; avoidance of pretend play) reflect that they still face some of the same difficulties in terms of socio-emotional development. This suggests that problems in interactions are not solely related to DHH children’s hearing capacity but are a consequence of multiple factors, such as the context in which interactions occur, the type of peers available, and the sensitivity/awareness of TH peers towards the DHH child. In this sense, environmental changes (e.g., changes in the playground setting) should accompany the progress of technology in providing these children with better hearing so as to increase their social participation. Nowadays, most DHH children are integrated into mainstream education, and interventions in this setting are needed to promote closeness and positive social interactions with their TH peers. Interventions should increase the awareness of TH children on what is needed to communicate with a DHH child (i.e., the child has to see your face, for example, in order to also use lip reading or to see where the sound is coming from), promoting more, and better quality interactions. Furthermore, exercise play seems to be a promising tool for promoting interactions between TH and DHH children; therefore, interventions through play within the peer group (e.g., psychomotor therapy) can play an important role in DHH children’s inclusion. Future research should address the impact of exercise play on the social inclusion of DHH children.

## Figures and Tables

**Table 1:** Mean scores (SD), mean rank and results of Friedman’s Rank Text for children’s engagement in different social levels, activity, and types of play by group.

	DHH Group	TH Group
	Mean (SD)	Mean (SD)
*Social Level*		
Social **	0.57 (0.19)	0.75 (0.16)
Parallel	0.00 (0.01)	0.01 (0.03)
Nonsocial **	0.42 (0.18)	0.24 (0.16)
*-Unoccupied*	*0.11 (0.08)*	*0.09 (0.08)*
-Onlooking **	*0.11 (0.07)*	*0.06 (0.07)*
-Solitary *	*0.20 (0.15)*	*0.09 (0.09)*
Not Observed	0.01 (0.02)	0.00 (0.01)
	χ^2^(2) = 18.67 ^1,^**	χ^2^(2) = 152.83 ^1,^**
*Activity*		
Play	0.44 (0.17)	0.42 (0.19)
Games with rules	0.02 (0.05)	0.08 (0.15)
Communication	0.23 (0.17)	0.28 (0.17)
Exploratory Behavior	0.02 (0.04)	0.03 (0.06)
Transition	0.02 (0.02)	0.02 (0.03)
Personal Care	0.02 (0.04)	0.01 (0.02)
Aggressive Behavior	0.00 (0.01)	0.00 (0.01)
Other Activities	0.02 (0.03)	0.01 (0.02)
Reticent	0.22 (0.11)	0.15 (0.12)
Not Observed	0.01 (0.03)	0.00 (0.01)
	χ^2^(8) = 77.40 ^2,^**	χ^2^(8) = 429.33 ^2,^**
*Type of Play*		
Exercise *	0.52 (0.29)	0.32 (0.28)
Rough-and-Tumble	0.13 (0.18)	0.15 (0.22)
Fantasy Play	0.02 (0.06)	0.12 (0.22)
Role Play	0.08 (0.17)	0.15 (0.21)
Constructive Play	0.05 (0.14)	0.06 (0.15)
Equipment	0.19 (0.15)	0.17 (0.25)
Other	0.01 (0.03)	0.03 (12)
	χ^2^(6) = 31.71 **	χ^2^(6) = 89.10 **

^1^ Including the Nonsocial, Parallel, and Social categories; ^2^ Excluding the “Not Observed” category; * *p* < 0.05; ** *p* < 0.01.

**Table 2:** Wilcoxon pairwise comparisons regarding types of activity DHH/TH children.

	Play	Games W. Rules	Communication	Exploratory	Transition	Personal C.	Aggressive	Other
Games with rules	−3.06 **/ −7.09 ***	-	-	-	-	-	-	-
Communication	−1.96/ −3.84 ***	−3.06 **/ −5.87 ***	-	-	-	-	-	-
Exploratory	−3.06 **/ −7.82 ***	−0.51/ −1.65	−3.06 **/ −7.90 ***	-	-	-	-	-
Transition	−3.06 **/ −7.94 ***	−0.87/ −1.93	−3.06 **/ −7.77 ***	−0.12/ −0.59	-	-	-	-
Personal C.	−3.06 **/ −7.91 ***	−0.54/ −3.84 ***	−3.06 **/ −7.93 ***	−0.32/ −2.9 **	−0.92/ −3.86 ***	-	-	-
Aggressive	−3.06 **/ −7.91 ***	−1.07/ −4.17 ***	−3.06 **/ −7.96 ***	−0.94/ −4.03 ***	−2.38 */ −4.48 ***	−0.73/ −2.02 *	-	-
Other	−3.06 **/ −7.91 ***	0/ −3.71 ***	−3.06 **/ −7.97 ***	−0.42/ −3.08 **	−0.41/ −4.34 ***	−0.32/ −0.55	−1.84/ −0.82	-
Reticent	−2.75 **/ −6.81 ***	−3.06 **/ −3.66 ***	−0.55 **/ −4.42 ***	−3.06 **/ −6.79 ***	−3.06 **/ −6.87 ***	−3.06 **/ −7.43 ***	−3.06 **/ −7.62 ***	−3.06 **/ −7.43 ***

* *p* < 0.05; ** *p* < 0.01; *** *p* < 0.001.

**Table 3:** Wilcoxon pairwise comparisons regarding types of play of DHH/TH children.

	Exercise	RTP	Fantasy	Role	Constructive	Equipment
Rough-and-Tumble	−2.67 **/ −4.05 ***	-	-	-	-	-
Fantasy Play	−2.98 **/ −4.18 ***	−1.68/ −0.83	-	-	-	-
Role Play	−2.51 */ −3.71 ***	−0.98/ −0.30	0.85/ −1.31	-	-	-
Constructive	−2.75 **/ −5.58 ***	−1.26/ −2.77 **	−1/ −2.03 *	−0.68/ −3.41 **	-	-
Equipment	−2.35 **/ −3.42 **	−1.25/ −0.55	−2.50 */ −1.15	−1.58/ −0.19	−1.8/ −3.15 **	-
Other	−2.98 **/ −6.56 ***	−1.72/ −4.51 ***	−0.73/ −3.47 **	−1.99 */ −4.75 ***	−0.73/ −1.27	−2.81 **/ −4.32 ***

* *p* < 0.05; ** *p* < 0.01; *** *p* < 0.001.

## Data Availability

The data that supports the findings of this study are available from the corresponding author upon reasonable request.
